# Asymmetrical Macular Thinning on Optical Coherence Tomography (OCT) in Pigmented Paravenous Retinochoroidal Atrophy

**DOI:** 10.7759/cureus.95746

**Published:** 2025-10-30

**Authors:** Anaam Rahman, Anahita Jamil

**Affiliations:** 1 Department of Ophthalmology, Rawal Institute of Health Sciences, Islamabad, PAK; 2 Department of Ophthalmology, Northwest General Hospital and Research Center, Peshawar, PAK

**Keywords:** fundus autofluorescence, macular thinning, optical coherence tomography, pigmented paravenous retinochoroidal atrophy, retinal dystrophy

## Abstract

Pigmented paravenous retinochoroidal atrophy (PPRCA) is a rare, typically non-progressive retinal disorder characterized by perivenous atrophy of the retinal pigment epithelium and choriocapillaris, with macular involvement being uncommon. This report aims to highlight a novel manifestation of asymmetrical macular thinning in PPRCA. A 40-year-old man presented with bilateral, gradual visual decline. Fundus examination revealed perivenous retinochoroidal atrophy with pigmentation along the arcades in both eyes and notable macular changes. Spectral-domain optical coherence tomography (OCT) demonstrated bilateral central retinal thinning, most marked in the temporal parafovea, with attenuation of both inner and outer retinal layers and preservation of the foveal depression. Fundus autofluorescence showed corresponding areas of hypoautofluorescence, while systemic evaluation excluded infectious and inflammatory causes. This case, to our knowledge, represents the first description of asymmetrical macular thinning in PPRCA, broadening the recognized spectrum of its macular involvement and emphasizing the diagnostic value of OCT in differentiating PPRCA from inflammatory and inherited retinal disorders.

## Introduction

Pigmented paravenous retinochoroidal atrophy (PPRCA) is a rare, typically bilateral retinal disorder characterized by perivenous atrophy of the retinal pigment epithelium (RPE), choriocapillaris, and overlying neurosensory retina. Most patients are asymptomatic or have mild visual loss, and fundus examination shows characteristic pigmentation and atrophy along the retinal veins [1]. Although its pathogenesis remains uncertain, both post-inflammatory and genetic factors have been implicated. Mutations in the CRB1 gene have been reported in some cases, linking PPRCA to a wider spectrum of CRB1-associated retinal dystrophies [2,3].

Optical coherence tomography (OCT) findings in PPRCA have mainly shown outer retinal and RPE thinning, choroidal attenuation, and occasional cystoid macular edema [4,5]. Other reported changes comprise star-shaped exudates, macular wrinkling, epiretinal membranes (with or without foveoschisis), macular pigmentary alterations such as stippling or depigmentation, lamellar and full-thickness macular holes, RPE atrophy, and even excavated macula in advanced stages. Despite these observations, significant macular involvement remains uncommon, and asymmetrical macular thinning has not been previously well documented [6].

Reporting atypical imaging features such as asymmetrical macular thinning is important because it expands the recognized spectrum of PPRCA and assists in differentiating it from post-inflammatory retinopathies and CRB1-related dystrophies. This report describes a case of bilateral PPRCA with asymmetric macular thinning on OCT, highlighting its clinical and diagnostic relevance.

## Case presentation

A 40-year-old man presented with a progressive, painless decrease in vision over five months. He denied nyctalopia, photophobia, systemic inflammatory disease, infectious exposures, or use of retinotoxic medications. There was no family history of retinal dystrophy or consanguinity. He had no history of prior ocular trauma or surgery.

Best-corrected visual acuity was 6/36 in both eyes. Anterior segment examination was unremarkable, and intraocular pressures were 12 mmHg bilaterally. Amsler grid testing was attempted but was unreliable due to poor patient cooperation, limiting interpretation.

Right eye

**Figure 1:**
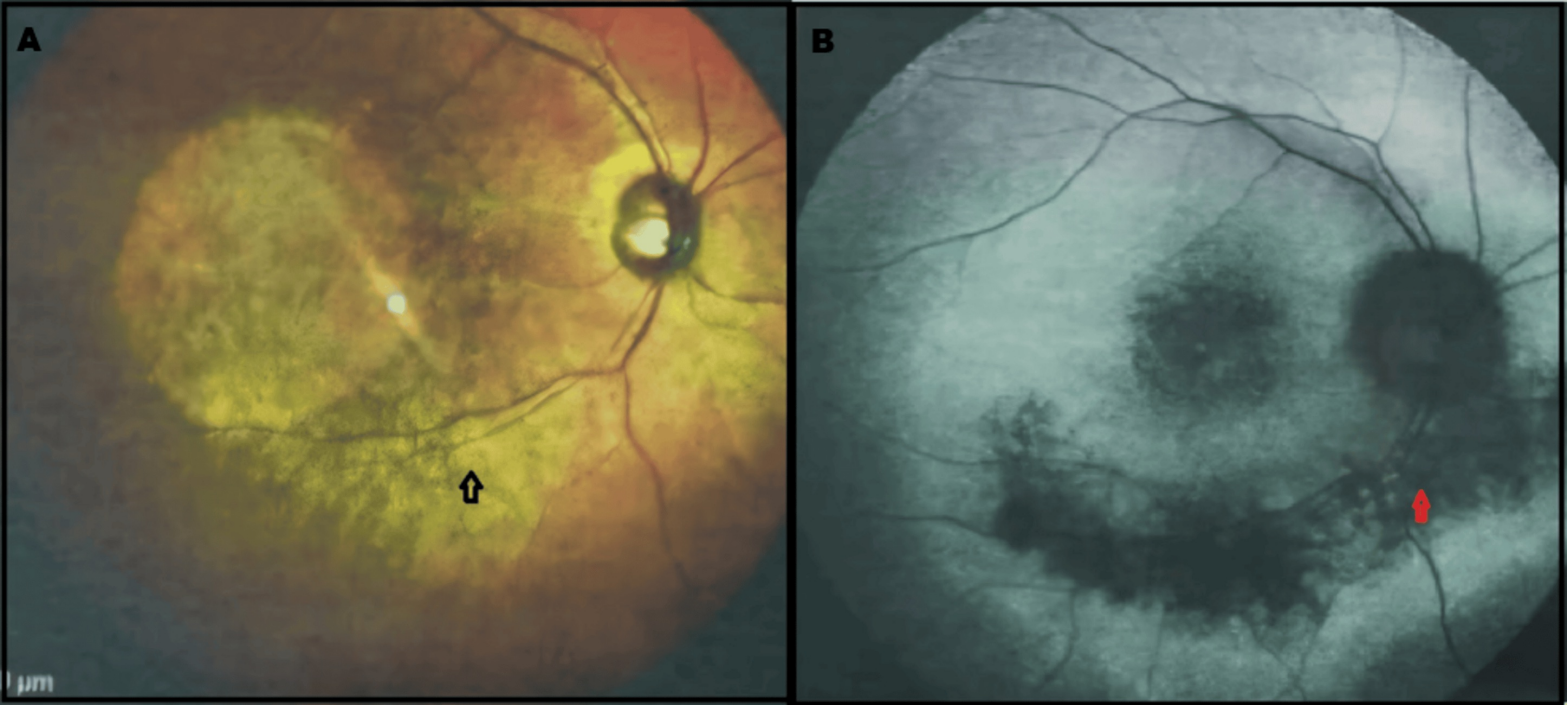
Color fundus photograph and corresponding fundus autofluorescence image of the right eye (A, B) Color fundus photograph demonstrating retinochoroidal atrophy along the inferior arcade (black) and macula, with corresponding zones of hypoautofluorescence (red arrow) on autofluorescence imaging. Image taken at Rawal Institute of Health Sciences. Overall image quality is limited by the available resolution

Fundus examination revealed perivenous retinochoroidal atrophy with pigment clumping predominantly along the inferior vascular arcades, which were more extensively involved than the superior arcades, extending toward the macular region. Mild arteriolar attenuation was also observed (Figure 1).

Left eye

**Figure 2:**
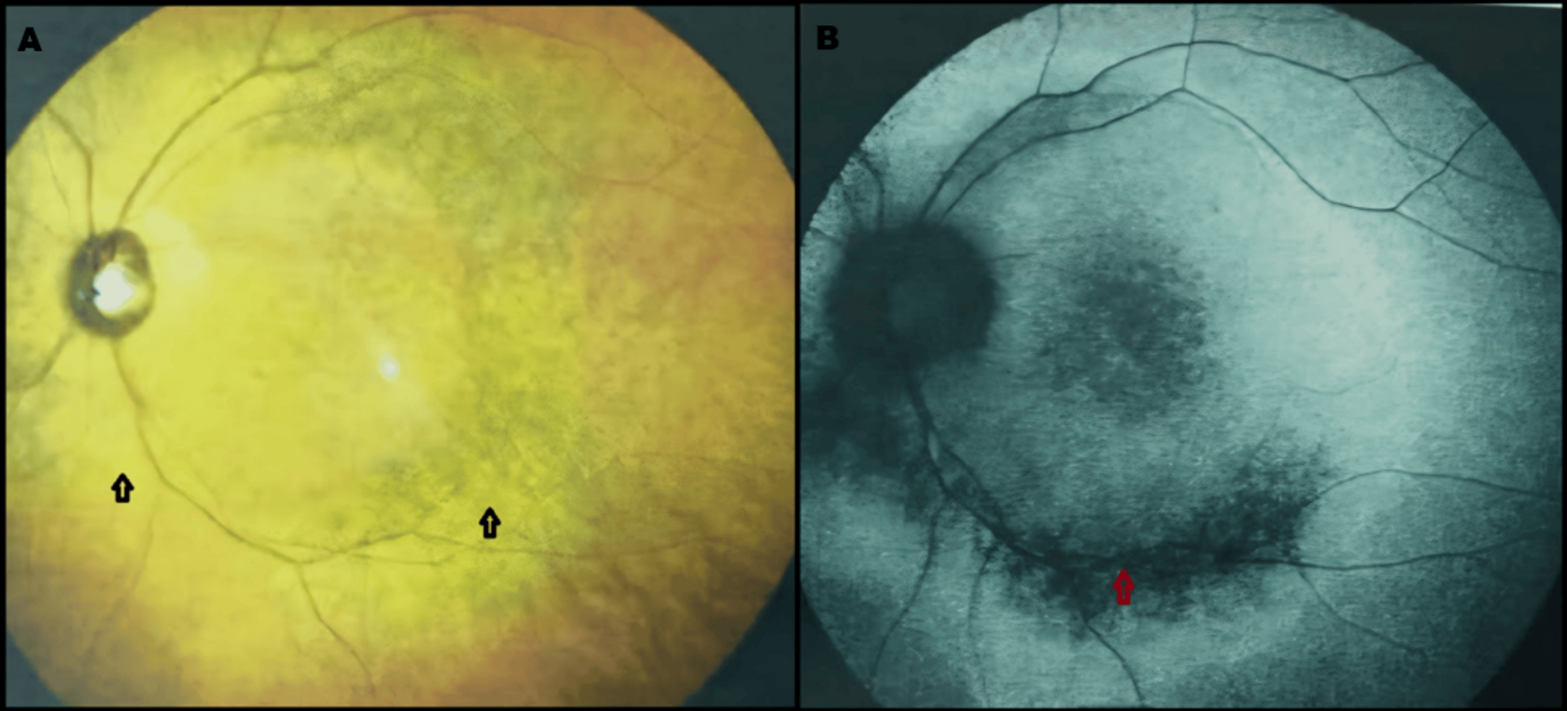
Color fundus photograph and corresponding fundus autofluorescence image of the left eye (A, B) Color fundus photograph demonstrating areas of retinochoroidal atrophy along the inferior arcade (black arrow) and macula, with corresponding zones of hypoautofluorescence (red arrow) on fundus autofluorescence imaging. Image taken at Rawal Institute of Health Sciences. Overall image quality is limited by the available resolution

Similar perivenous atrophic changes were observed along both the superior and inferior vascular arcades near the optic disc, with extension involving the macular region (Figure 2).

OCT findings

**Figure 3:**
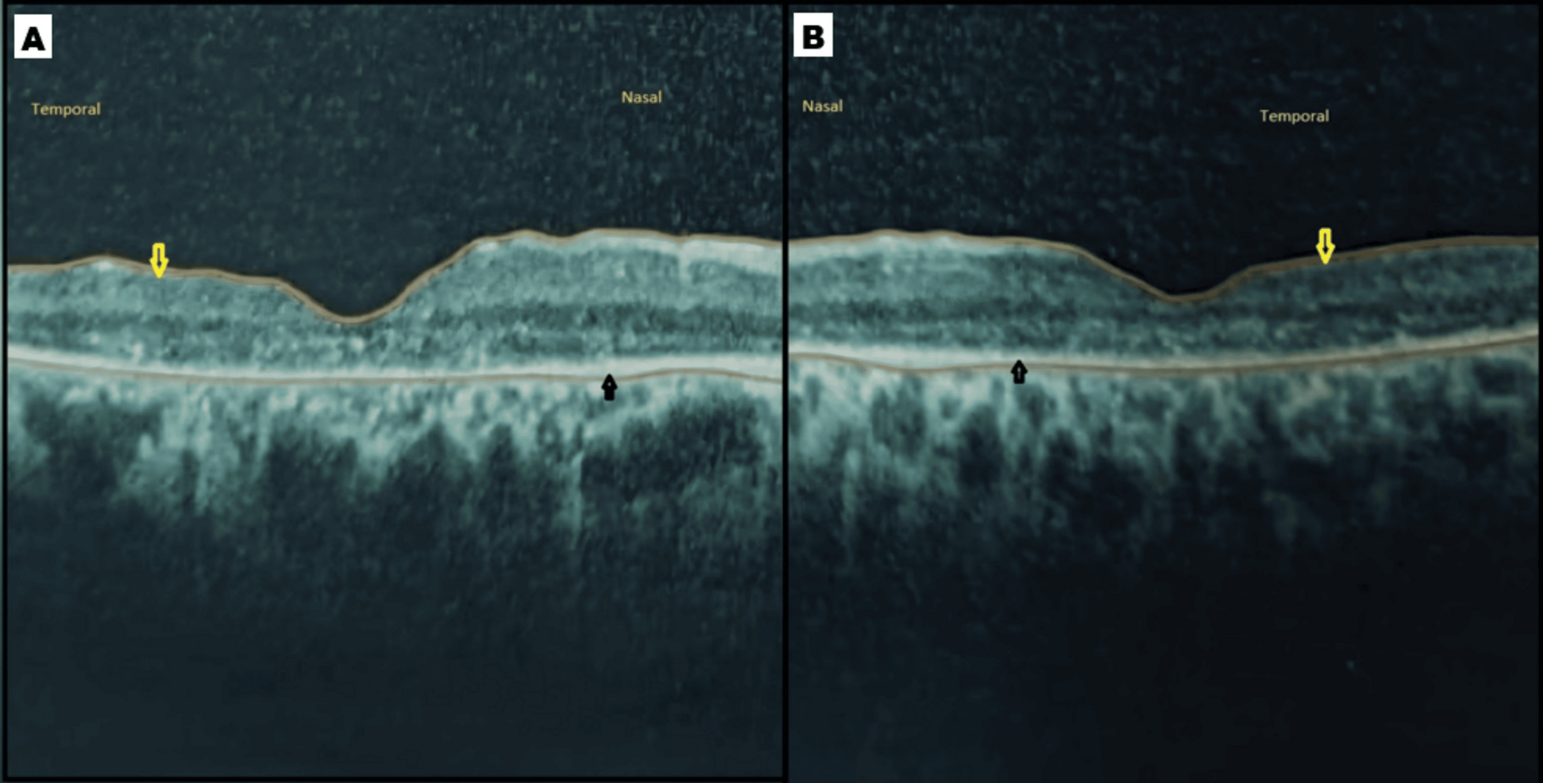
SD-OCT macula of both eyes (A, B) SD-OCT showing the right eye and left eye macula demonstrating thinning of the inner and outer retinal layers, more pronounced temporally (yellow arrow), while the RPE remains intact (black arrow). Image taken at Rawal Institute of Health Sciences SD-OCT: spectral-domain optical coherence tomography

Spectral-domain OCT demonstrated bilateral central thinning, more marked temporally than nasally, with thinning and disruption of both inner and outer retinal layers. Foveal contour was preserved, and the internal limiting membrane showed mild undulation (Figure 3).

Fundus autofluorescence

Hypoautofluorescent arcs corresponding to areas of atrophy were seen in both eyes.

A comprehensive systemic evaluation (including tuberculosis, syphilis, sarcoidosis, measles, herpes simplex virus (HSV), and toxoplasmosis testing) was negative. The patient was counseled about the chronic, non-inflammatory, and likely progressive nature of the condition.

## Discussion

PPRCA is a rare and often incidentally detected condition characterized by perivenular pigment clumping and adjacent chorioretinal atrophy [1]. Its first description dates back to 1937, when Brown reported a case of chorioretinal atrophy confined to the immediate proximity of the retinal veins, which he termed “retinochoroiditis radiata” [7].

More recent molecular insights have highlighted a genetic contribution, particularly involving mutations in the CRB1 gene. These mutations disrupt photoreceptor-Müller cell polarity and adhesion, giving rise to a phenotypic spectrum that includes PPRCA, retinitis pigmentosa, and Leber congenital amaurosis [2,3]. The dual inflammatory and genetic hypotheses are not mutually exclusive; environmental or inflammatory insults may unmask disease in genetically predisposed individuals.

Macular thinning, as observed in our patient, represents a finding not specifically documented in the existing literature. Earlier angiographic studies suggested that primary RPE dysfunction may precede choroidal vascular involvement, whereas newer OCT and OCT angiography studies indicate that choroidal thinning and choriocapillaris attenuation can occur even before detectable RPE changes. Both human and animal models have shown that thinning of the outer and inner nuclear layers can occur while the RPE remains relatively intact [6]. In contrast, as reported by Khan et al., in CRB1-associated maculopathy, OCT shows outer retinal and macular atrophy, intraretinal cysts in inner and outer nuclear layers (INL, ONL), initial sparing of foveola, and progressive perifoveal outer retinal atrophy, often resulting in a bull’s-eye maculopathy pattern and varying degrees of retinal thickening with preserved choroidal thickness [8]. The post-infectious or inflammatory forms of PPRCA have very limited macular data reported in the literature. Ramtohul et al. described a case of PPRCA associated with Vogt-Koyanagi-Harada disease presenting with cystoid macular edema [9]. In our case, OCT demonstrated thinning of the inner and outer retinal layers, while the RPE remained relatively intact, suggesting early neuroretinal degeneration.

The pathogenesis of PPRCA remains debated, with both inflammatory and genetic mechanisms proposed. Reported inflammatory and infectious associations include Vogt-Koyanagi-Harada disease, presumed ocular tuberculosis, syphilis, and viral infections such as Epstein-Barr virus, cytomegalovirus, and HSV [9-12].

In the present case, the absence of prior infection, intraocular inflammation, or positive serology supports a dystrophic rather than post-inflammatory origin. The asymmetrical macular thinning involving inner and outer retinal layers with preserved RPE and somewhat foveal represents a potentially novel structural phenotype within the PPRCA spectrum. This highlights the evolving role of OCT in identifying subtle retinal changes that might otherwise go undetected.

Clinically, recognizing these OCT patterns is essential for differentiating PPRCA from post-inflammatory chorioretinal atrophy and CRB1-related dystrophies, which may have overlapping fundus appearances but distinct systemic and genetic implications. A thorough systemic and infectious work-up remains crucial before attributing paravenous atrophy to a hereditary etiology.

In summary, this case expands the phenotypic spectrum of PPRCA, documenting asymmetrical macular thinning with preserved RPE on SD-OCT. It underscores the value of multimodal imaging in distinguishing dystrophic from inflammatory causes and highlights the need for genetic evaluation in atypical or asymmetric presentations.

## Conclusions

This case highlights asymmetrical macular thinning as a novel structural manifestation in PPRCA. Spectral-domain OCT demonstrated bilateral involvement, with more pronounced thinning in the temporal parafovea, affecting both inner and outer retinal layers, while the foveal contour remained reserved but not entirely normal. Clinically, the patient experienced a gradual, painless visual decline without signs of intraocular inflammation or systemic disease. These findings expand the recognized phenotypic spectrum of PPRCA and underscore the value of multimodal imaging in characterizing subtle macular changes, which may inform patient counseling and follow-up strategies.
